# Supplementary material for: Importance of the PD-1/PD-L1 Axis for Malignant Transformation and Risk Assessment of Oral Leukoplakia
Source: Biomedicines. 2021 Feb 16;9(2):194. doi: 10.3390/biomedicines9020194 (PMC7920045; doi:10.3390/biomedicines9020194)
Supplement: Supplementary file 1 [file biomedicines-09-00194-s001.pdf]

biomedicines

Supplementary Material

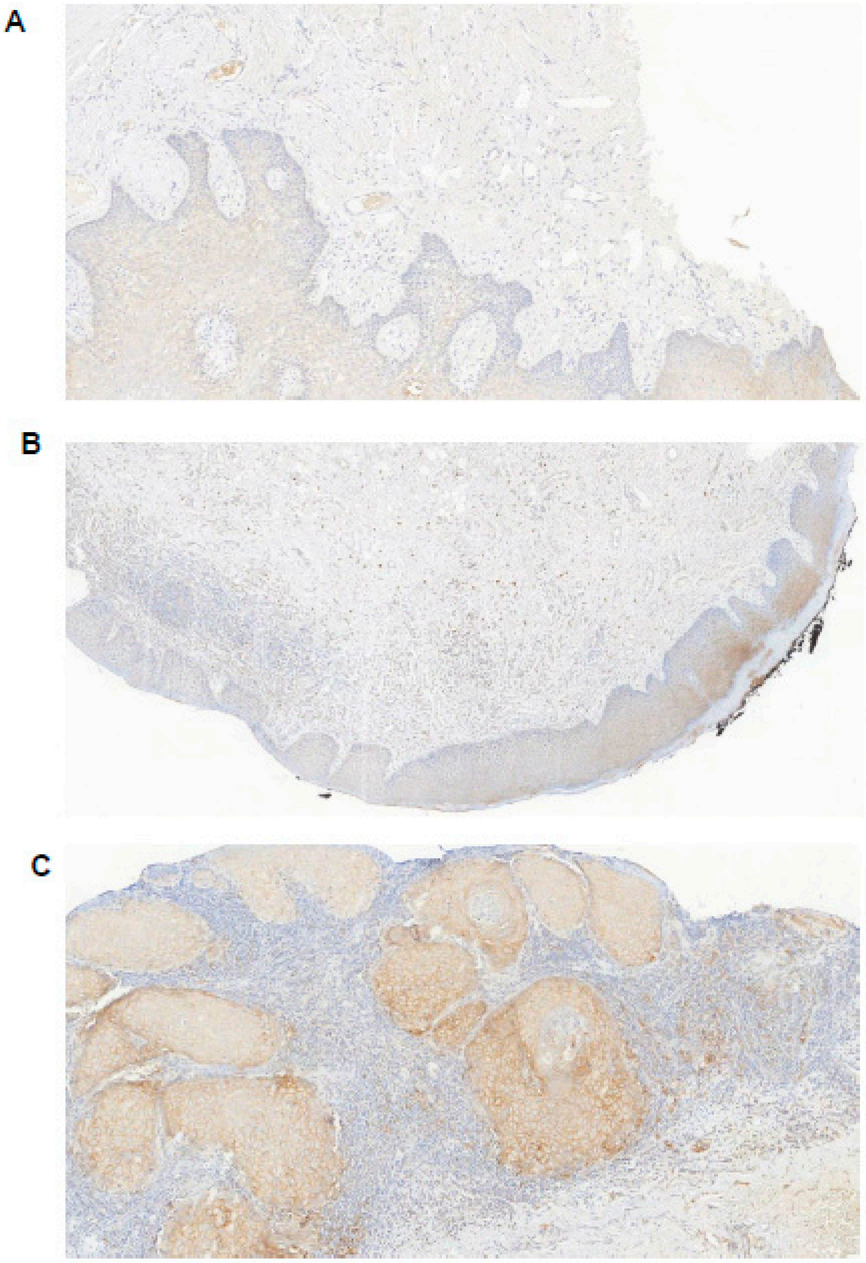

Figure S1. Classification "none", "mild", "moderate" and "severe" inflamed samples according to infiltration density by inflammatory cells in the subepithelium of the affected tissue (sections stained applying antibody against PD-L1). (**A**) mild (**B**) moderate (**C**) severe. (magnification 10×).
